# The impact of social network use on adolescent depression: the chain mediation between self-objectification and body satisfaction

**DOI:** 10.3389/fpsyg.2024.1347858

**Published:** 2024-05-27

**Authors:** Yiming Tang, Min Xu, Zhongwei Tan, Yong Liu

**Affiliations:** ^1^School of Psychology, South China Normal University, Guangzhou, China; ^2^Experimental Center of Psychology, South China Normal University, Guangzhou, China

**Keywords:** social network use, self-objectification, body satisfaction, depression, chain mediation

## Abstract

**Introduction:**

Adolescents are in the transitional stage from childhood to adulthood, a critical period for individual physical and mental development. With the rapid development of the Internet, social networking has become an integral part of adolescents’ daily lives. However, the information that adolescents are exposed to on social networks is often processed and embellished, which may cause them to become physically dissatisfied and lead to emotional problems, such as depression. We investigated the chain-mediating effects of self-objectification and body satisfaction on the relationship between social network use and depression.

**Methods:**

We utilized questionnaire data of 2025 adolescents from two secondary schools and one high school in China.

**Results:**

Our results demonstrated that (1) there are obvious sex differences in the intensity of social network use and active and passive social network use among adolescents, with usage higher among girls than for boys; (2) self-objectification and body satisfaction play a mediating role in the relationship between the intensity of social network use and adolescent depression, as well as the presence of chain-mediating roles; and (3) self-objectification and body satisfaction play an intermediary role in the relationship between active and passive social network use and adolescent depression; there is further a chain intermediary role. The findings suggest that social network use affects adolescents’ depression through self-objectification and body satisfaction, which is not only manifested in the general use intensity of social networks but also in their active and passive use modes of social networks.

**Conclusion:**

This study provides theoretical support for the causes and mechanisms behind the influence of social network use on adolescent depression and has practical implications for the prevention and intervention of adolescent emotional problems.

## 1 Introduction

Adolescence is a critical period for individuals’ physical, mental, and social development, and a dangerously confusing stage for individuals coping with various contradictions and conflicts. If adolescents do not cope properly with various negative life events, they are prone to negative subjective experiences and psychological problems (Lei et al., 2003).

Depression usually refers to a negative emotion or serious mood disorder in an individual. Researchers generally view depression as a mind state involving emotions such as displeasure, low spirit, and distress, in which the individual is more pessimistic, slower to act, slower to react, and lacks interest (Lin et al., 2003). Depression is significantly negatively correlated with an individual’s body satisfaction (Stice et al., 2000; Roberts and Duong, 2013).

Body satisfaction, also known as body image satisfaction, is an individual’s evaluation of his or her body. Body image dissatisfaction is often defined as a negative evaluation of one’s own body, including the perceived discrepancy between one’s assessment of the actual body and ideal body (Szymanski and Cash, 1995). Body dissatisfaction may be an influential factor in depression (Ferreiro et al., 2014). Further, a depressed state of mind can occur with low body satisfaction (Fung and Yuen, 2003). Body satisfaction is subject to sociocultural influences in the same way as other subjective perceptions and experiences.

Thompson et al. (1999) proposed a three-pronged model of influence based on sociocultural theory, also known as the tripartite influence model. This theoretical model assumes that there are three sociocultural factors–family, peers, and mass media–that influence body image. These lead to body dissatisfaction through appearance comparison and internalization of “ideal thinness.” The model provides a comprehensive account of social and cultural influences on individual body image, influences which have been confirmed in numerous studies (Olivardia et al., 2004; Fardouly and Vartanian, 2016).

Social network use is used to describe individuals who can use functions and enjoy the services provided by social network platforms by registering on social network accounts and logging in as users. For example, people can chat and make friends online, publish their own photos and remarks, interact with friends, and obtain news (Boyd and Ellison, 2007; Han, 2020). Social network use generally refers to the intensity of social network use and can be divided into active social network use and passive social network use according to usage patterns (Burke et al., 2010). Active social network use refers to the activity of individuals who engage in direct communication, such as making remarks, posting their own photos, liking and commenting on their friends’ updates, or chatting in private messages on social platforms. Contrastingly, passive social network use refers to activities in which individuals simply browse friends’ homepages or newsfeeds on social media platforms without direct communication (Burke et al., 2010; Verduyn et al., 2015).

Many studies have explored the relationship between social network use and depression; however, no consistent conclusions have emerged. A subset of studies found a significant positive correlation between social network use and depression (Ding et al., 2016; Liu et al., 2018), demonstrating that individuals who spend more time on social networks have higher negative emotions as compared to their counterparts (Fardouly et al., 2015). However, some researchers have also argued that there is no obvious correlation between social network use and depression and that the appropriate use of social networks can effectively alleviate depression (Yao et al., 2014). One of the main reasons for these disputes may be the lack of delineation of social network use behavior. Active social network use can enhance interpersonal communication and emotional exchange, which is conducive to reducing individuals’ loneliness and depression (Burke et al., 2010; Deters and Mehl, 2013; Chen et al., 2016; Ding et al., 2017). Passive social network use is essentially browsing large amounts of “positively biased” information from social networks. During browsing, a large amount of information inevitably triggers users to engage in upward social comparison behaviors, leading to negative and depressive emotions (Zhu et al., 2021).

According to the tripartite influence model (Thompson et al., 1999), media is a main source of sociocultural factors. Compared to traditional mass media, social networks are more communicative and interactive, in which social pressure mainly comes from socially proximate others, such as family members, friends, and companions. When individuals are under pressure to meet a certain body image set by these socially proximate others, they will internalize the esthetic standards in social networks, such as “thin” and “high,” as the evaluation criteria of their body image. These internalizations lead to unsatisfactory evaluations of their bodies and cause negative emotions (Fardouly et al., 2015; Tiggemann and Zaccardo, 2015; Hendrickse et al., 2017; Rousseau et al., 2017).

Bartky (1990) first proposed the concept of sexual objectification, in which women’s bodies and sexual functions are regarded as “tools.” When experiencing sexual objectification, women’s bodies will be perceived as existing to please and serve others. Fredrickson and Roberts (1997) developed the objectification theory, which holds that women’s bodies are often scrutinized, evaluated, and potentially viewed as objects; this is the sexual objectification experience that women encounter. The process of self-objectification occurs when women accept the gaze of others; that is, they internalize this gaze and view their bodies through it. This process of self-objectification is followed by a range of negative psychological or subjective experiences that lead to psychological or behavioral symptoms, such as eating disorders, depression, and sexual dysfunction. Several factors affect the degree of an individual’s self-objectification. Among these, sexually objectifying messages on television (Aubrey, 2006) and in music videos (Aubrey and Frisby, 2011) increase the extent of female self-objectification. With the rise of new media, cyberspace has become filled with sexually objectifying messages. Social networks subtly promote an ideal standard of beauty that does not conform to objective reality, leading users to unnecessary esthetic introspection with greater exposure. The more time and energy women spend on social media, the more likely they are to scrutinize themselves against the harsh esthetic standards promoted on social media, and thus, the easier it is to experience negative emotions such as appearance dissatisfaction (Aubrey, 2006; Hatton and Trautner, 2011; Frisby and Aubrey, 2012). However, different ways of using social networks may have different effects on self-objectification. For example, browsing other people’s photos, publishing one’s own photos, and other specific social network use behaviors may have a more direct impact on women’s sense of self-objectification (Meier and Gray, 2014).

In summary, according to the tripartite influence model (Thompson et al., 1999), on the one hand, social network use can affect an individual’s degree of self-objectification, leading to negative emotions of depression. On the other hand, social network use can also affect an individual’s body satisfaction. Owing to the mismatch between the actual-body and the ideal-body image standard, depressive emotions are generated. In addition, individuals will evaluate their body images during the process of self-objectification, thereby affecting their body satisfaction. Therefore, social network use can also indirectly affect an individual’s depressive mood.

**Figure 1:**
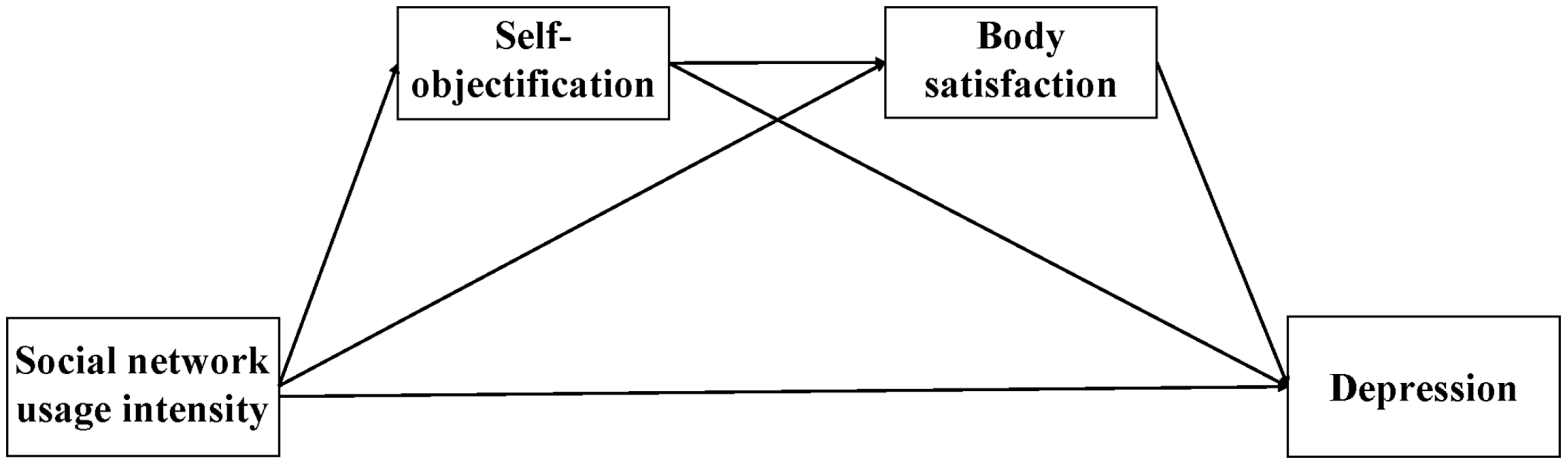
The influence of active and passive social network use on depression: chain mediation model hypothesis diagram.

**Figure 2:**
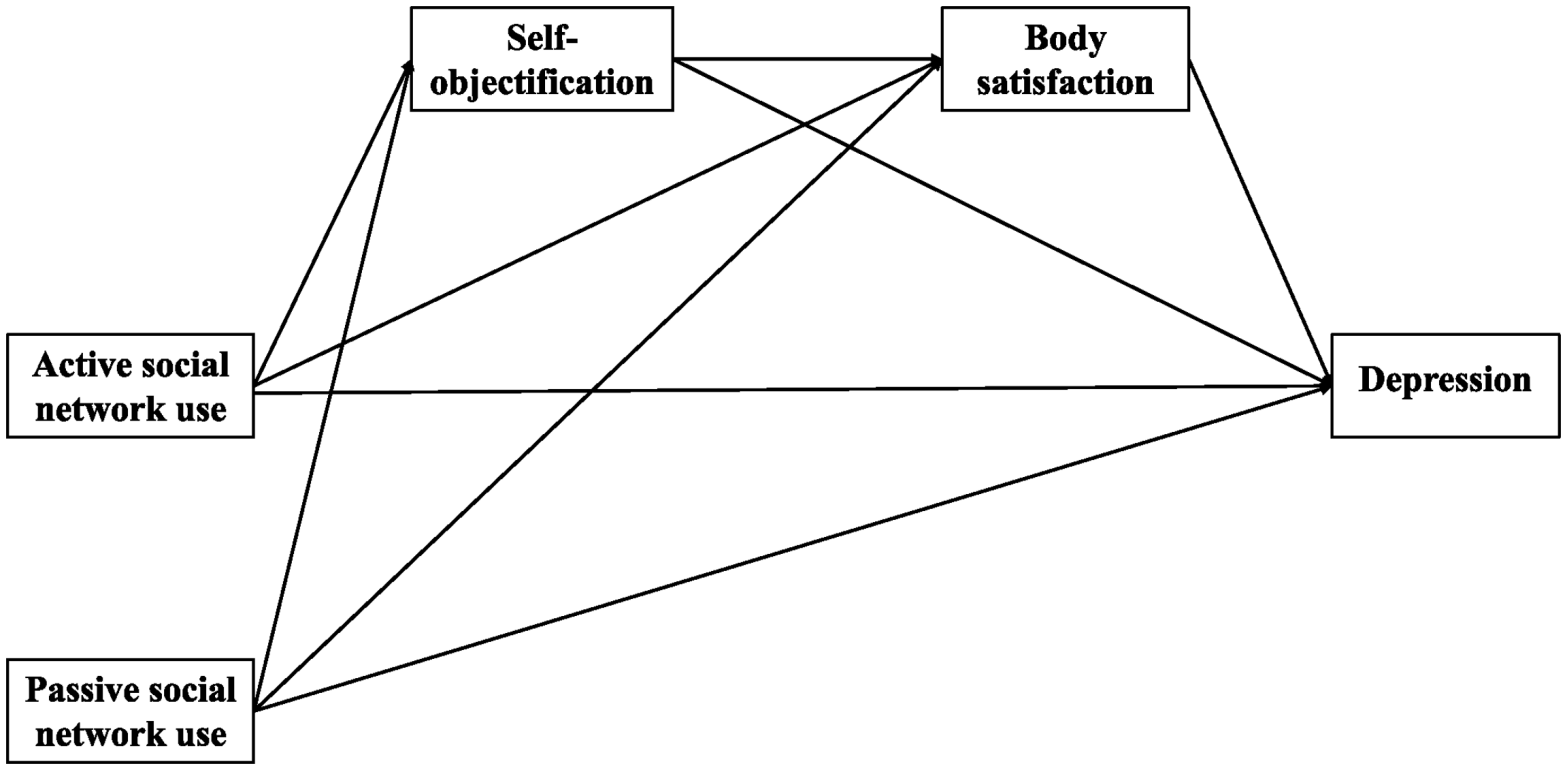
The path coefficient of the effect of social network on depression.

Based on the above discussion, we constructed a chain mediation model (Figures 1, 2) to explore the impact of social network use (general social network use intensity and different social network use styles) on adolescent depression and its internal mechanism, the mediation of self-objectification, and body satisfaction.

## 2 Methods

### 2.1 Participants

We used convenience sampling to select 1,258 junior high school students and 1,027 senior high school students from two ordinary secondary schools (one junior high school and one senior high school) in a city in southern China. We obtained 2025 valid questionnaires after deleting omitted and regularly answered (chose the same answer to every question) questionnaires. Among them, 1,007 (49.7%) were boys and 1,018 (50.3%) were girls; 530 (26.2%) were only children and 1,477 (72.9%) were not; 503 (24.8%) were in the first grade of junior high school, 693 (34.2%) were in the second grade of junior high school, 403 (19.9%) were in the first grade of senior high school, and 426 (21.0%) were in the second grade of senior high school. The mean age was 14.68 ± 1.551 years (range = 11–18 years).

### 2.2 Measures

#### 2.2.1 Social networking usage intensity

We used the Chinese version of the Intensity of Social Network Use Questionnaire developed by Ellison et al. (2007) and translated by Niu et al. (2016a), which comprises eight items. The first two items measure the number of friends an individual has on a social network site and their average daily usage time. The last six items measure the degree of dependence on social networks and the extent of individuals’ emotional involvement. The response options range from 1 = “very unconformable” to 5 = “very conformable.” As the scoring methods of the first two questions differ from those of the last six questions, the final score of the scale was calculated by standardizing the scores of all the questions, adding them together, and then calculating the average. Higher scores represent a higher intensity of individual social network use. This scale is reliable and valid (Jiang et al., 2019; Jiang and Wang, 2021). Cronbach’s alpha was 0.75 in this study, indicating acceptable internal consistency.

#### 2.2.2 Active social network use

The Active SNS Use Scale developed by Frison and Eggermont (2015) was adopted with five items. The response options range from 1 (*never*) to 5 (*always*). It primarily measures an individual’s active use of networking sites. For example, “updating information on one’s own homepage,” “posting photos on one’s own homepage,” and so on. This scale is reliable and valid (Lian et al., 2017; Chen et al., 2021). In this study, the scale was unidimensional, and the synthetic reliability of the scale was calculated using SPSS 25.0. The result was 0.73. Cronbach’s alpha was 0.80, indicating good internal consistency.

#### 2.2.3 Passive social network use

The Passive SNS Use Scale developed by Tandoc et al. (2015) was revised by Liu et al. (2017) and comprises four questions. The response options range from 1 (*never*) to 7 (*multiple times per day*). It mainly measures individuals’ passive use of social networks, such as “reading the updated status of friends” and “viewing photos uploaded by friends.” This scale is reliable and valid (Li et al., 2022; Zhang and Zeng, 2023). In this study, the scale was one-dimensional, and the synthetic reliability of the scale was calculated using SPSS 25.0. The result was 0.69. Cronbach’s was 0.86, indicating good internal consistency.

#### 2.2.4 Self-objectification

We used the Objectified Body Consciousness Scale compiled by Lindberg et al. (2006) and revised in Chinese by Chen and Jiang (2007). The scale comprises eight questions and is scored on a seven-point scale: 1 (*completely inconsistent*) to 7 (*completely consistent*). Two items are scored normally (e.g., “I often worry about whether the clothes I wear make me look good”) and six items are reverse-scored (e.g., “For me, it is more important to wear comfortable clothes than whether they look good” and “I seldom compare my appearance with others”). This scale is reliable and valid (Zhang and Zhong, 2018). Higher scores indicate higher levels of self-objectification. Cronbach’s α was 0.79 in this study, indicating acceptable internal consistency.

#### 2.2.5 Body satisfaction

We adopted the Multidimensional Body-Self Relations Questionnaire, developed by Cash (2000) and revised by Wang and Wang (2004) in Taiwan. The scale includes nine items, each rated on a five-point scale 1 (*strongly disagree*) to 5 (*strongly agree*). This scale is reliable and valid (Guo and Chen, 2015; Nie and Wang, 2015). Higher scores indicated higher body satisfaction. Cronbach’s alpha was 0.87 in this study, indicating good internal consistency.

#### 2.2.6 Depression

The Center for Epidemiological Studies Depression Scale-20, compiled by Radloff (1977) and revised by Chen et al. (2009), was used to ask individuals to report their depressive feelings or states of mind during the previous week. This scale comprises 20 items. The response options range from 1 (*less than 1 day*) to 4 (*5–7 days*). This scale is reliable and valid (Liu et al., 2023; Wang and Zhao, 2023). Higher scores indicate more severe depression. Cronbach’s alpha was 0.90 in this study, indicating excellent internal consistency.

### 2.3 Procedure

This study was approved by the Ethics Review Committee of the School of Psychology at South China Normal University (no. SCNU-PSY-2022-157). Participants provided their written informed consent to participate. Based on the relevant contents of the ethical review, junior and senior high schools in Foshan City, Guangdong Province, were selected to complete the questionnaire survey. Paper questionnaires were used, with scales randomly arranged during the questionnaire preparation process. This resulted in three different versions to reduce the influence of scale position on the test results. The test was administered in class using uniform instructions and standardized testing procedures. The primary examiners were trained graduate students majoring in psychology. Before the test, the main examiners read the informed consent form to participants and explained the purpose and significance of the survey, the scientific nature of the research, and the confidentiality of the results. The questionnaires were completed anonymously and were distributed and collected immediately.

### 2.4 Data analyses

All data were entered, sorted, and saved using SPSS 25.0. Descriptive statistics, difference tests, correlation analyses, and intermediary model tests were made using SPSS 25.0 and PROCESS plug-ins.

## 3 Results

### 3.1 Preliminary analyses

The descriptive statistics and zero correlations of all variables are shown in Table 1. The intensity of active social network use and passive social network use were positively correlated with self-objectification and depression and negatively correlated with body satisfaction. Self-objectification negatively correlated with body satisfaction and positively correlated with depression. Body satisfaction was negatively correlated with depression.

**Table 1:** Descriptive statistics and zero-order correlations of variables.

	*M*	*SD*	1	2	3	4	5	6	7	8	9	10
1. Sex	1.50	0.50										
2. Grade	2.37	1.07	0.01	1								
3. Age	14.68	1.55	−0.01	0.93^**^	1							
4. Body mass index	19.44	3.00	−0.09^**^	0.16^**^	0.15^**^	1						
5. Social network use	5.13	2.51	0.165**	0.032	0.019	−0.017	1					
6. Active network use	2.81	0.812	0.16^**^	0.14^**^	0.10^**^	−0.05^*^	0.182^**^	1				
7. Passive network use	4.09	1.18	0.16^**^	0.15^**^	0.13^**^	−0.01	0.57^**^	241^**^	1			
8. Self-objectification	3.72	1.07	0.28^**^	0.11^**^	0.10^**^	−0.04^*^	0.26^**^	0.29^**^	0.290^**^	1		
9. Body satisfaction	3.10	0.70	0.22^**^	−0.14^**^	−0.12^**^	−0.13^**^	−0.07^**^	−0.07^**^	−0.38^**^	−0.064^**^	1	
10. Depression	1.83	0.54	0.14^**^	0.00	−0.01	−0.01	113^**^	0.12^**^	0.32^**^	−0.37^**^	0.46^**^	1

Body mass index is calculated as weight (kg)/height (*m*)^2^. The average value of “social network usage intensity” is the average value of the last six items of the scale, and the standardized score is used for correlation analysis. *p < 0.05; **p < 0.01; ***p < 0.001.

### 3.2 The serial mediation model between social network use and depression

Model 6 of the PROCESS macro (Hayes, 2013) was used to test the mediating role of self-objectification and body satisfaction, controlling for sex, age, and body mass index. As shown in Table 2, social network use significantly and positively predicted depression (β = 0.136, *p* < 0.001), self-objectification (β = 0.393, *p* < 0.001), and body satisfaction (β = 0.069, *p* < 0.05). Further, self-objectification negatively predicted body satisfaction (β = −0.325, *p* < 0.05) and positively predicted depression (β = 0.184, *p* < 0.05). Body satisfaction was negatively correlated with depression (β = −0.277, *p* < 0.05). Moreover, the direct effects of social network use on depression were not significant (β = 0.048, *p* > 0.05) after controlling for the impact of self-objectification and body satisfaction.

**Table 2:** Results of the successive mediated model between social network use and depression.

	Effect size	Boot SE	95% CI		Relative effect
			Lower	Upper	
Indirect effect					
SNU → SO → DEP	0.072	0.012	0.050	0.097	80.90%
SNU → BS → DEP	−0.019	−0.009	−0.038	−0.001	−21.35%
SNU → SO → BS → DEP	0.035	0.005	0.026	0.046	39.33%
Total indirect effect	0.089	0.017	0.056	0.121	100%

SNU, social network use; SO, self-objectification; DEP, depression; BS, body satisfaction.

**Figure 3:**
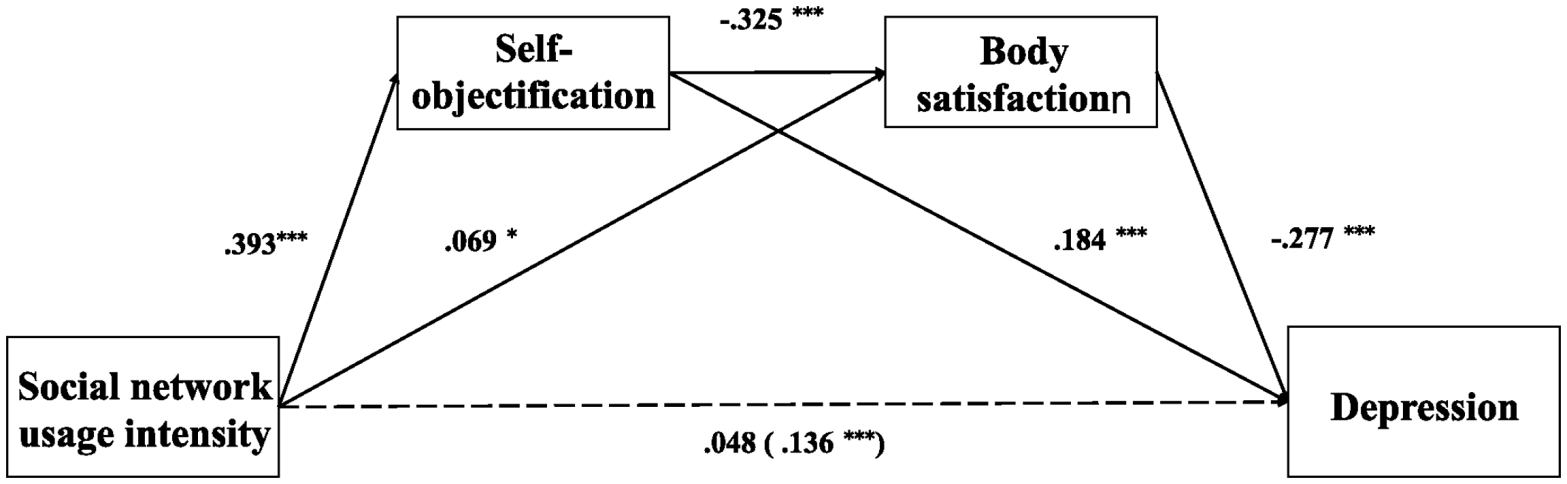
The path coefficient of the impact of active and passive social network on depression.

Concerning indirect effects, the pathway of “social network use → self-objectification → depression” was significant [indirect effect = 0.072, 95% CI = (0.050, 0.097)]. The indirect effect of social network use on depression through body satisfaction was significant [indirect effect = −0.019, 95% CI = (−0.038, −0.001)]. In addition, the indirect effect of social network use on depression through self-objectification and body satisfaction was significant [indirect effect = 0.035, 95% CI = (0.026, 0.046)]. Further, the total indirect effect was significant [indirect effect = 0.089, 95% CI = (0.056, 0.121)]. These results show that self-objectification and body satisfaction partially and successively mediated the association between social networking use and depression (Figure 3).

### 3.3 The serial mediation model of active and passive social network use on depression

The PROCESS macro (Model 6) was adopted to examine the successive mediating roles of self-objectification and body satisfaction in active and passive social network use and depression, controlling for sex, age, and body mass index.

As shown in Table 3, active social network use significantly and positively predicted depression (β = 0.058, *p* < 0.001), self-objectification (β = 0.198, *p* < 0.001), and body satisfaction (β = 0.038, *p* < 0.05). Self-objectification was negatively related to body satisfaction (β = −0.322, *p* < 0.05) and positively related to depression (β = 0.190, *p* < 0.05). In addition, body satisfaction was significantly and negative related to depression (β = −0.277, *p* < 0.05). Further, the direct effects of active social network use on depression were not significant (β = 0.048, *p* > 0.05) after controlling for the impact of self-objectification and body satisfaction.

**Table 3:** The serial mediation model of active social network use on depression.

	Effect size	Boot SE	95% CI		Relative effect
			Lower	Upper	
Indirect effect					
ASNU → SO → DEP	0.038	0.007	0.026	0.051	65.52%
ASNU → BS → DEP	−0.011	0.006	−0.022	−0.001	−18.97%
ASNU → SO → BS → DEP	0.018	0.003	0.012	0.024	31.03%
Total indirect effect	0.045	0.010	0.026	0.064	77.59%

ASNU, active social network use; SO, self-objectification; DEP, depression; BS, body satisfaction.

The indirect effect of active social network use on depression through self-objectification was significant [indirect effect = 0.038, 95% CI = (0.026, 0.051)]. The pathway of “active social network use → body satisfaction → depression” was significant [indirect effect = −0.011, 95% CI = (−0.022, −0.001)]. The indirect effect of active social network use on depression through self-objectification and body satisfaction was successively significant [indirect effect = 0.018, 95% CI = (0.012, 0.024)]. In addition, the total indirect effect was not significant [indirect effect = 0.045, 95% CI = (0.026, 0.064)]. These results indicate that self-objectification and body satisfaction successively mediated the association between active social network use and depression.

As shown in Table 4, passive social network use was positively correlated to depression (β = 0.093, *p* < 0.05), self-objectification (β = 0.235, *p* < 0.001), and body satisfaction (β = 0.051, *p* < 0.05). Self-objectification was negatively associated with body satisfaction (β = −0.326, *p* < 0.05), and positively associated with depression (β = 0.181, *p* < 0.05). In addition, body satisfaction was significantly and negative related to depression (β = −0.278, *p* < 0.05). Further, the direct effects of active social network use on depression was also significant (β = 0.044, *p* < 0.05) after controlling for impacts of self-objectification and body satisfaction.

**Table 4:** The serial mediation model of passive social network use on depression.

Effect	Effect size	Boot SE	95% CI		Relative effect
			Lower	Upper	
Indirect effect					
PSNU → SO → DEP	0.043	0.007	0.029	0.058	45.74%
PSNU → BS → DEP	−0.014	0.006	−0.027	−0.003	−14.89%
PSNU → SO → BS → DEP	0.021	0.003	0.016	0.028	22.34%
Total indirect effect	0.050	0.010	0.030	0.070	53.19%

PSNU, passive social network use; SO, self-objectification; DEP, depression; BS, body satisfaction.

**Figure 4:**
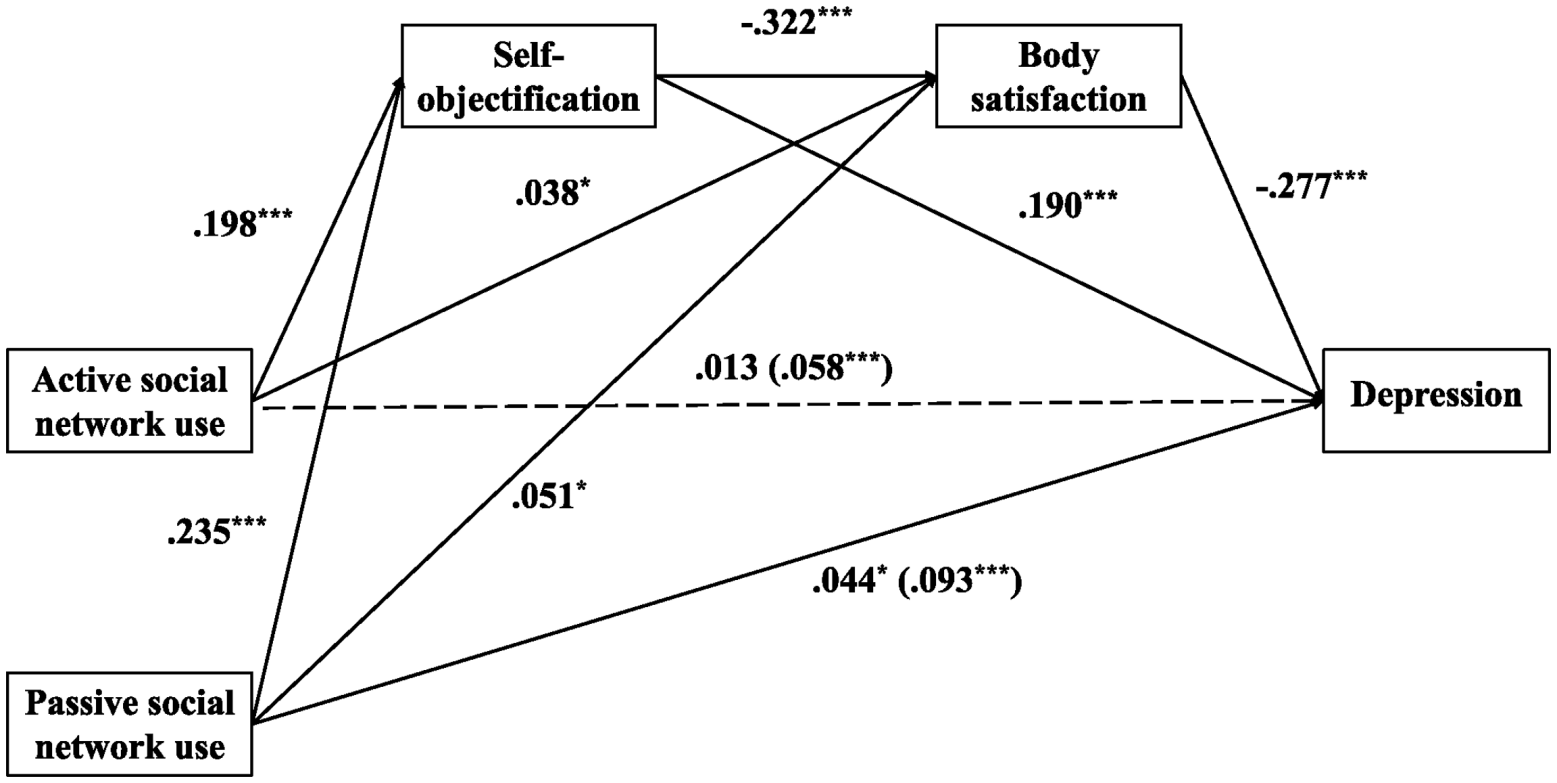
The influence of social network usage intensity on depression: chain mediation model hypothesis diagram.

Concerning indirect effects, the pathway of “passive social network use → self-objectification → depression” was significant [indirect effect = 0.043, 95% CI = (0.029, 0.058)]. The indirect effect of passive social network use on depression through body satisfaction was significant [indirect effect = −0.014, 95% CI = (−0.027, −0.003)]. In addition, the indirect effect of passive social network use on depression through self-objectification and body satisfaction was successively significant [indirect effect = 0.021, 95% CI = (0.016, 0.028)]. Further, the total indirect effect was significant [indirect effect = 0.050, 95% CI = (0.030, 0.070)]. These results demonstrate that self-objectification and body satisfaction partially and successively mediated the relationship between passive social network use and depression (Figure 4).

## 4 Discussion

### 4.1 The impact of social network use to depression: the mediating role of self-objectification and body satisfaction

Social network use was a positive predictor of depression. Previous studies have confirmed that the time spent on social networks is positively correlated with negative emotions (Fardouly et al., 2015). Greater social network use in adolescents may lead to a greater risk for depression (Jelenchick et al., 2013; Frison and Eggermont, 2015, 2016). The current results further confirm this conclusion and extend it to Chinese adolescents.

According to the three-aspect influence model of social culture (Thompson et al., 1999), social networks are important for social and cultural dissemination. Social networks provide a platform for people to share their lives. The large number of ideal and beautiful images presented on social networks lead users to make more body comparisons, resulting in body dissatisfaction and depression. From the perspective of objectification theory, users may experience self-objectification when browsing and interacting with pictures and videos on social networks. Consequently, social networks may make users overly concerned about their appearance and figure, further increasing the risk of body dissatisfaction and depression. To further explain this phenomenon, we introduced self-objectification and body satisfaction and found that they played a complete mediating role in the association between social network use and depression.

The current results enrich objectification theory and the three-aspect influence model of social culture. On the one hand, people post pictures, videos, and other self-information on social media to receive objective evaluations from friends or strangers. On the other hand, when people browse other people’s body information on social media and provide negative or positive reactions, such as through comments and likes, this becomes a process of unconscious objectification of others. According to a study on social media use, as the intensity of social media use increased, women scored higher on body surveillance and body shame as compared to men. Further, specific social media usage behaviors, such as browsing other people’s photos and posting one’s own photos, had a more direct impact on women’s self-objectification consciousness as compared to men (Moradi and Huang, 2008; Meier and Gray, 2014). According to the objectification theory, a core symptom of self-objectification is continuous body monitoring (Lindner et al., 2012). When individuals continuously monitor their bodies, self-objectification makes them adopt a negative attitude toward recognizing their bodies, which negatively affects their body satisfaction. The less satisfied individuals were with their bodies, the more likely they were to experience depression. Body dissatisfaction plays an important mediating role in the relationship between self-objectification and depression (Mitchell and Mazzeo, 2009).

### 4.2 The impact of active and passive social network use on depression: the mediating role of self-objectification and body satisfaction

We found a correlation between active and passive social network usage. Active and passive social network use have significant positive predictive effects on depression. This result is inconsistent with prior results. Some researchers believe that active and passive social network use are independent of each other and have completely different effects (Verduyn et al., 2015). Active social network use can strengthen daily contact and emotional communication and promote offline communication (Deters and Mehl, 2013). Active social network use can strengthen individuals’ social communication abilities and reduce loneliness and depression (Ellison et al., 2007). Although active social interactions may provide emotional support to individuals, they are still exposed to a large amount of negative information. Similarly, the possibility of social comparison in the process of active social interaction cannot be ignored as it may lead to anxiety and inferiority, thereby increasing the risk of depression. Passive social network use may reduce an individual’s happiness and significantly predict depression (Burke et al., 2010; Chen et al., 2016; Ding et al., 2017). However, some scholars have confirmed that depression is the cause of passive social network use among college students (Zhu et al., 2021). Overall, the relationship between active and passive social network use and depression is complex and requires further investigation.

To further study the intermediate mechanism, we introduced self-objectification and body satisfaction and found that they played a complete mediating role between active social network use and depression, whereas they played a partial mediating role between passive social network use and depression. Both active and passive social network usage patterns remained significant in this model, supporting our hypothesis. However, self-objectification and body satisfaction play a completely intermediate role between active social network use and depression, which demonstrates that among various factors that lead to depression, the possibility of depression occurring owing to body image is critical. Many adolescents actively initiate communication, create and transmit information, and obtain social support and emotional communication through their social networks. However, in this process, some self-presentation behaviors (such as posting selfies), social interaction behaviors (e.g., comments), and even the exchange of objectified information may expose individuals to an objectified environment, leading to higher self-objectification, body dissatisfaction, and depression.

Self-objectification and body satisfaction played partially intermediate roles in the relationship between passive social network use and depression, suggesting that other factors may play a role in the effect of passive social network use on depression. Self-esteem, enjoyment, rumination, and other variables can play a mediating role in the relationship between passive social network use and depression (Tandoc et al., 2015; Appel et al., 2016; Niu et al., 2016b; Zhang and Zhou, 2018).

### 4.3 Limitations and prospects for future research

This study had some limitations that should be addressed in future research. First, this study verified the influence of social networks on depression but did not consider individual differences. Several demographic factors play an important role in the development of depression, such as education, marriage, and economic status. There are many other factors that can influence adolescent depression, such as personality type, family caregiving, self-esteem, and so on, and more variables can be used to produce more comprehensive and practically meaningful results in future studies. By exploring the interactions and combined effects of various factors, a more holistic understanding of the complex nature of adolescent depression can be achieved.

An individual’s self-esteem, personality, and motivation may play important roles in this process. For example, in the current field of cyberpsychology and body image research, many researchers have proposed the potential protective effect of social media literacy on the negative effect of exposure to appearance-oriented social media images on body satisfaction, which can alleviate the negative body image brought about by contact with idealized images on social media (McLean et al., 2013, 2016; Wilksch and Wade, 2015; Mahon and Hevey, 2021). Media literacy means that individuals can control the potential negative impact on body image by actively questioning the purpose of content presentation and understanding the authenticity of images (Burnette et al., 2017; Evens et al., 2021). Future studies should consider the mediating role of media literacy and other individual differences in the relationship between social networking use and body image.

The intensity and pattern of social network have a positive predictive effect on depression, but the relationship between the two is complex, and scholars have different views. One of the main reasons for these disputes may be the lack of classification of social network use behaviors (Frison and Eggermont, 2015; Tandoc et al., 2015). Most results show a significant positive correlation between social network use and depression (Fardouly et al., 2015); however, in some situations, social network use can enhance social communication. Increased daily contact and emotional interaction may promote offline activities and reduce feelings of loneliness and depression (Deters and Mehl, 2013). The relationship between social network use and depression is influenced by multiple factors; thus, more exploration is needed.

Although this study confirmed that social network use can influence body dissatisfaction through self-objectification, and then lead to depression, the relationship between the two is complex. Previous findings have been inconsistent. Some note that social media use is associated with negative body image and negative mood (Holland and Tiggemann, 2016), while others have found that social media use is associated with positive body image (Marika et al., 2020). This may involve more careful categorization of social media. A meta-analysis of cross-sectional relationships found a significant but small positive association between social media use and body dissatisfaction, especially appearance-focused social media use (Saiphoo and Vahedi, 2019). Empirical studies on social media sites such as Instagram and Facebook found that the use of social media can positively predict body image worries and lead to negative emotions and body dissatisfaction (Tiggemann et al., 2018; Prichard et al., 2021). Therefore, the relationship between different types of social media and body dissatisfaction and depression requires elucidation. Some of the more visually oriented social platforms may be associated with body dissatisfaction and depression, and we speculate that the reason for this may be that users are more likely to view photos and post selfies on them.

The adoption of a self-evaluation approach may lead to biased results. Adolescents may under- or over-report their social media use, self-objectification experiences, or mental health symptoms, leading to inaccuracies in the data and subsequent findings. Future studies should seek to employ more objective and reliable measurement methods. For instance, researchers could consider using third-party tools or applications to monitor social media usage objectively. Additionally, clinical interviews conducted by trained professionals could be employed to assess mental health symptoms more accurately. By combining subjective self-reports with objective measures, future studies can aim to minimize bias and enhance the validity of their findings.

The use of social networks is a real behavior or behavioral tendency, and more real-world data presentation may be needed to improve the ecological effect of research. There are various ways to measure self-objectification and body satisfaction. For example, body satisfaction can be measured using image selection, figure-drawing tests, behavioral observation, and computer experiments. Self-objectification includes an experimental manipulation paradigm, objectified word priming, and viewing ideal-body videos (Roberts and Gettman, 2004; Harper and Tiggemann, 2008; Tiggemann and Boundy, 2008). Future studies may be more in-depth and comprehensive if other measurement methods and experimental paradigms are used. These could include eye movement detection, electroencephalography, and some physiological indicators.

Finally, the sample was recruited from only two schools in a province in southern China, and the ecological validity of the results may be low. Therefore, the results cannot be easily generalized to northern China or other cultural contexts. Future studies should aim to recruit a larger, more diverse sample and perform cross-cultural validation studies.

## 5 Conclusion

This study tested the relationship between social network use and depression, along with the mediating effects of self-objectification and body satisfaction. The results demonstrated that (1) social network use was a positive predictor of depression; (2) self-objectification and body satisfaction sequentially mediated the relationship between social network use and depression; and (3) self-objectification and body satisfaction separately and successively mediated the relationship between active and passive social network use and depression. These results contribute to the understanding of the relationship between these two variables and their underlying mechanisms.

## Data availability statement

The original contributions presented in the study are included in the article/Supplementary material, further inquiries can be directed to the corresponding author.

## Ethics statement

The studies involving humans were approved by Ethics Review Committee of the School of Psychology at South China Normal University (No. SCNU-PSY-2022-157). The studies were conducted in accordance with the local legislation and institutional requirements. Written informed consent for participation in this study was provided by the participants’ legal guardians/next of kin.

## Author contributions

YT: Conceptualization, Methodology, Writing – original draft. MX: Data curation, Formal analysis, Writing – review & editing. ZT: Resources, Visualization, Writing – review & editing. YL: Supervision, Writing – review & editing.
